# Characterisation of *HvVIP1* and expression profile analysis of stress response regulators in barley under *Agrobacterium* and *Fusarium* infections

**DOI:** 10.1371/journal.pone.0218120

**Published:** 2019-06-14

**Authors:** Nadia El Sarraf, Filiz Gurel, Feyza Tufan, Liam J. McGuffin

**Affiliations:** 1 Department of Agriculture and Food Engineering, University of Balamand, Koura, Lebanon; 2 Department of Molecular Biology and Genetics, Istanbul University, Istanbul, Turkey; 3 Institute of Science, Program of Molecular Biology and Genetics, Istanbul University, Istanbul, Turkey; 4 School of Biological Sciences, University of Reading, Whiteknights, Reading, Berkshire, United Kingdom; Consiglio per la Ricerca e la Sperimentazione in Agricoltura, ITALY

## Abstract

*Arabidopsis thaliana’s* VirE2-Interacting Protein 1 (VIP1) interacts with *Agrobacterium tumefaciens* VirE2 protein and regulates stress responses and plant immunity signaling occurring downstream of the Mitogen-Activated Protein Kinase (MPK3) signal transduction pathway. In this study, a full-length cDNA of 972bp encoding *HvVIP1* was obtained from barley (*Hordeum vulgare* L.) leaves. A corresponding 323 amino acid poly-peptide was shown to carry the conserved bZIP (Basic Leucine Zipper) domain within its 157^th^ and 223^rd^ amino acid residue. 13 non-synonymous SNPs were spotted within the *HvVIP1* bZIP domain sequence when compared with *AtVIP1*. Moreover, minor differences in the bZIP domain locations and lengths were noted when comparing *Arabidopsis thaliana* and *Hordeum vulgare* VIP1 proteins through the 3D models, structural domain predictions and disorder prediction profiling. The expression of *HvVIP1* was stable in barley tissues infected by pathogen (whether *Agrobacterium tumefaciens* or *Fusarium culmorum*), but was induced at specific time points. We found a strong correlation between the transcript accumulation of *HvVIP1* and barley PR- genes *HvPR1*, *HvPR4* and *HvPR10*, but not with *HvPR3* and *HvPR5*, probably due to low induction of those particular genes. In addition, a gene encoding for a member of the barley MAPK family, *HvMPK1*, showed significantly higher expression after pathogenic infection of barley cells. Collectively, our results might suggest that early expression of PR genes upon infection in barley cells play a pivotal role in the *Agrobacterium*-resistance of this plant.

## Introduction

*Agrobacterium tumefaciens* genetically transforms cells by stably integrating a fragment of its extrachromosomal DNA plasmid, the T-DNA, into the plant genome [1]. Molecular and genetic changes occurring within the host organism during the transfection process remain relatively unknown and little is understood about the host factors involved in the process [2–5]. In the model organism *Arabidopsis thaliana*, the host effector VIP1 interacts with the pathogen’s virulence VirE2 protein (VIP1: VirE2-Interacting Protein 1) and its associated T-complex inside the cell cytosolic space, and guides them into the cell nucleus via its Nuclear Localization Signal motif (NLS), where it then promotes T-DNA integration into the host genome [3, 6–9]. Therefore, VIP1 is thought to be employed by the pathogen to increase the rate of plant genetic transformation. However, VIP1 has been identified as a component of the mitogen-activated protein kinase (MAPK) signal transduction pathway, associated with the regulation of stress responses and plant immunity signalling [10,11]. The current authors attempt to explain and generate theoretical hypotheses and models which can reveal the reasons behind VIP1’s dual function and its controversial behaviours. Our colleagues [4] explained that, during transfection, both the host and *Agrobacterium* compete in promoting one or other of the two functions of VIP1; “plant susceptibility” to *Agrobacterium* infection is the determining factor in which role VIP1 actually performs. Thus, they confirmed Karami’s [12] claim that susceptibility to infection remains a genotype-related issue that might vary between tissues and cell types even within the very same plant [13]; more specifically, there may be a correlation with the expression level of certain host proteins [14]. Theoretically, throughout transfection, interaction between VIP1 and bacterial virulence proteins would probably lead the T-DNA to the promoters of particular stress-responsive genes regulated by VIP1; thus, the bacterium’s ability to hijack plant stress response for its own benefits has been highlighted [5,15]. VIP1 (also referred to as bZIP51) protein belongs to a large bZIP family (Basic Leucine Zipper) of transcription factors [16] and has been first characterized in *Arabidopsis thaliana* [7]. Recently, eight VIP1 homologs have been described in *Arabidopsis* and tobacco in relation to their interactions with VirE2 [9]. The VIP1 protein sequence has been predicted in wheat (Accession No. AHY03428.1), rice (XP_015618202.1), maize (NP_001141497.1) and barley (BAJ96285.1), but no in-depth study of the protein’s structural nor functional characteristics, in monocotyledonous, which do not host *Agrobacterium*, has been undertaken.

Pathogenesis-related (PR) genes encoding PR proteins confer pathogen-resistance in host plants. They are usually expressed in plant cells after pathogenic attack or exposure to abiotic stresses, as well as during seed germination, development and senescence [17]. Despite PR proteins being known to possess antifungal activity [18–20], their precise mode of function is still insufficiently understood. In barley, *HvPR1*, *HvPR3*, *HvPR4*, *HvPR5* and *HvPR10* were found to be induced at different levels following infection with *Fusarium culmorum* [21], as well as after *Agrobacterium* infection (as our results have shown). Over the years, studies of the PR1 gene have provided information regarding its roles in plant stress responsiveness. [10] have suggested an MPK3-VIP1-PR1 regulation model in *Arabidopsis thaliana* cells whereby AtVIP1 transcription factor is considered a direct target of the *Agrobacterium*-induced mitogen-activated protein kinase (MAPK) MPK3, and such activation of VIP1 by MPK3 will lead eventually to induction of PR1. These findings were subsequently disputed by Pitzschke *et al*. [11], who claimed that there is no direct interaction between VIP1 and PR1 in reality, suggesting that VIP1’s role in the induction of PR1 expression must occur indirectly. These conflicting results call for resolution through bioinformatic assays or in-vitro protein-DNA binding assays. The barley MAPK family, including 16 MPK proteins, have been characterized previously as key regulators of stress response [22].

The objective of this study was to characterize the VIP1 protein in barley (*Hordeum vulgare* L., a recalcitrant crop to *Agrobacterium*-mediated transformation) and to search for structural similarities to and differences from its already characterised *Arabidopsis* orthologue. Furthermore, we aimed at identifying HvVIP1’s role as a transcriptional regulator of the plant stress response. The expression profile of *HvVIP1* as well as other genes implicated in stress (i.e. *HvPRs* and *HvMPK1*) were examined in pathogen-treated (i.e. *Agrobacterium* and *Fusarium* infected) barley tissues. Furthermore, correlations were examined between *HvMPK1* induction and *HvVIP1* as well as between *HvVIP1* and *HvPRs*.

## Results and discussion

### Characterisation of VIP1 in *Hordeum vulgare* L.

**Fig 1:**
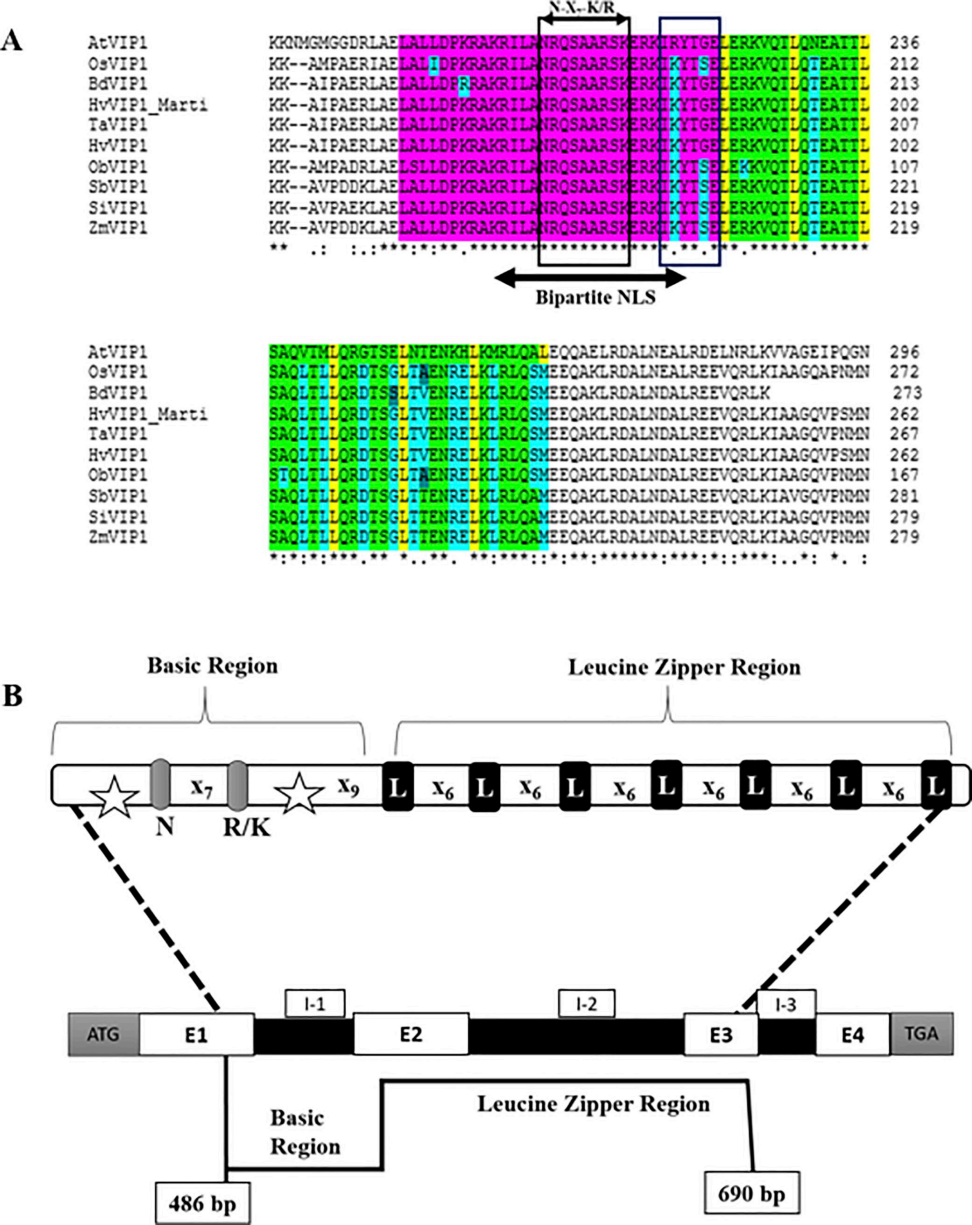
**(A) Alignment of the VIP1 bZIP domains of various homologous plant proteins identified by Blast search.** The bZIP domain of VIP1 (DDBJ/EMBL/GenBank accession No. NP_564486.1) was aligned (using the clustalW algorithm- EMBL-EBI/MUSCLE) with similar motifs of its homologues from *Oryza sativa* (OsVIP1), *Brachypodium distachyon* (BdVIP1), *Hordeum vulgare* (Both, predicted clone HvVIP1, accession No. BAJ96285.1 and our predicted protein sequence HvVIP1_Martı), *Triticum aestivum* (TaVIP1), *Oryza brachyantha* (ObVIP1), *Sorghum bicolor* (SbVIP1), *Setaria italica* (SiVIP1), *Zea mays* (ZmVIP1). Regions of identity are indicated as follows: (i) The basic region is highlighted in violet, (ii) the bipartite NLS within the basic region is denoted by a horizontal bar in grey below its sequence, (iii)the invariant N-X7-K/R motif location is indicated within the basic region, (iv) the Leucine Zipper region (seven Leucine heptad) is highlighted in yellow/green, (v) SNPs within the bZIP domains are marked individually and were identified according to AtVIP1. (*) indicates fully conserved residues, (:) and (.) indicate less conserved residues. **(B) Predicted gene model of *HvVIP1*.** The bZIP domain Basic region stretches over Exons 1 and 2 (E-1 and E-2). Within the basic region: bipartite NLS is marked in stars, and the invariant DNA binding motif (N-X7-R/K). The Leucine Zipper region is located on exons 2 and 3 (E-2 and E-3). I 1–3: Introns; (N): Asparagine; (R): Arginine; (K): Lysine; (L): Leucine; ATG: Start codon; TGA: Stop codon; X: any nucleotide.

**Fig 2:**
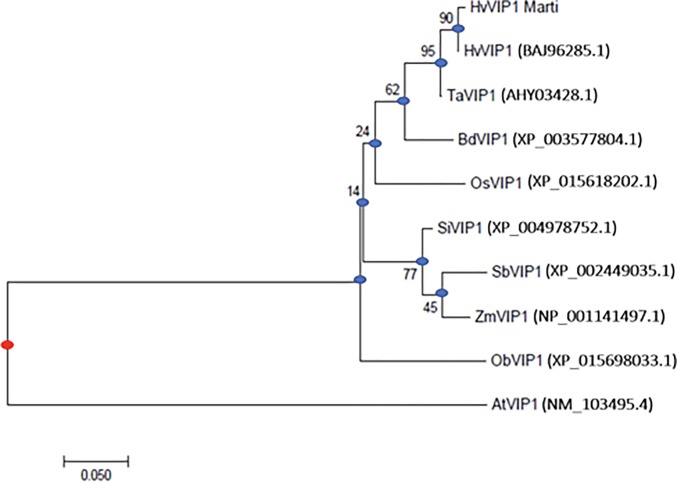
Phylogeny of the VIP1-like proteins. The multiple alignment was generated using MUSCLE software and the phylogenetic tree was built with the MEGA5 software using the JTT matrix-based model (the numbers at the nodes indicate the bootstrap scores). The protein accession numbers are indicated in parentheses. *Oryza sativa* (OsVIP1), *Brachypodium distachyon* (BdVIP1), *Hordeum vulgare* (Both, predicted clone HvVIP1, and our predicted protein sequence HvVIP1_Martı), *Triticum aestivum* (TaVIP1), *Oryza brachyantha* (ObVIP1), *Sorghum bicolor* (SbVIP1), *Setaria italica* (SiVIP1), *Zea mays* (ZmVIP1), and *Arabidopsis thaliana* (AtVIP1). Blue nodes are the putative ancestors; the red node represents the historical ultimate ancestor. The Bar (0.050) is a scale for the amount of genetic change (nucleotide substitutions per site).

The *HvVIP1* full length cDNA in *H*. *vulgare* L. cv. Martı is a 972 bp sized fragment encoding a protein of 323 amino acid residues (Accession No: MG000155). Sequence alignment with *Hordeum vulgare* cv. Haruna nijo predicted clone (DDBJ/EMBL/GenBank accession No. AK365082.1/ [23]) has identified four SNPs within the Martı cv. identified at the 115^th^, 204^th^, 872^nd^, and 896^th^ bp positions, respectively. The HvVIP1 protein sequence is of 323 amino acid residues containing a bZIP domain located near the protein’s C-Terminal Domain (CTD). Based on VIP1-like protein homologies and comparative analysis, the Basic Region (BR) of the bZIP domain was predicted to lie between the 157^th^ and the 187^th^ amino acid. It contains a Nuclear Localization Signal (NLS) sequence and an invariant DNA binding motif. The NLS consensus consists of two short stretches (bipartite) of basic amino acids, designated ^165^KRILANRQSAARSKERKIK^183^, which belongs most probably to the class six bipartite NLS model KR-X_10-12_-K(KR)-X-(K/R) [24]. The DNA binding domain is an invariant N-X_7_-R/K motif [16] designated within the HvVIP1 protein sequence as ^170^NRQSAARSK^178^, marked by the bZIP Group I characteristic Lysine (K) residue at the 178^th^ position replacing the usual Arginine (R) amino acid identified in all other bZIP protein family members. The hinge region located at the end of the Basic Region usually encompasses 6 amino acid residues. It is known for its additive effect upon protein DNA-binding specificity. The Leucine-Zipper (LZ) region is a conserved leucine heptad module located near the protein’s C-terminal domain. It is flanked by the 188^th^ and 223^rd^ residue, marking the end of the bZIP domain, when compared to AtVIP1 [11,16] (Fig 1A). Gene prediction analysis indicated that the HvVIP1 gene is located on the 4th chromosome and has 3620 bp (excluding UTRs). The analyses show that it contains 4 exons of 495 bp, 132 bp, 87 bp and 258 bp size respectively, intercalated by 3 introns of 215 bp, 1691 bp, and 148 bp-sized fragments, respectively. The bZIP domain stretches along Exon 1, 2, and 3, as indicated in the HvVIP1 gene model (Fig 1B). A phylogenetic tree was constructed using the complete amino acid sequences of VIP1-like bZIP proteins from a variety of monocotyledonous and dicotyledonous species. As a result, the VIP1 proteins from cereals were clustered into groups that each of them showed variation, while this clad was quite distinct from *AtVIP1* (Fig 2).

The Pfam server predicted a bZIP sequence domain between the 160^th^ and the 221^st^ residues within the HvVIP1 protein. In addition, the sub-sequences of the protein’s ordered regions, predicted using DISOclust, indicated that the ordered region containing the bZIP domain lies between the 146^th^ and the 257^th^ residues. Those results were confirmed with the IntFOLD server, which generated 3D models with high quality estimates, structural domain predictions, and disorder prediction profiles. The IntFOLD server indicated a likely bZIP ordered helical domain located between the 146^th^ and the 257^th^ amino acid residues (S1B Fig); whereas, in AtVIP1, the bZIP motif was registered, according to IntFOLD, between the 187^th^ and the 290^th^ amino acid, as shown in S1A Fig. These results suggest that the HvVIP1 Leucine Zipper region was likely to contain up to nine leucine heptad repeats. The leucines are sometimes substituted by isoleucine, valine, phenylalanine or methionine (e.g. Fig 1A- methionine replacing leucine at the 230th position). The intrinsic disorder prediction profile [25] (S1C Fig) of the full-length *H*. *vulgare* VIP1 predicted protein indicates that HvVIP1 is partially disordered at the N and C termini and has an ordered region spanning the146^th^ to the 257^th^ residues.

Overall, amino acid sequences of VIP1 orthologues retrieved from both dicotyledonous (*A*. *thaliana*) and monocotyledonous (barley, rice, wheat, maize, etc.) counterparts share certain structural similarities at the level of their ordered regions beholding the reputed bZIP domains [16, 26–28]. In our study, a variety of bioinformatics tools, domain database (i.e. Pfam), and servers (i.e. IntFOLD, DISOclust) were used to elucidate the structural features of *HvVIP1* protein and eventually to build a protein model [29,30]. IntFOLD server, an integrated web resource for protein fold recognition [31] provided 3D model quality assessment of the submitted protein, whereas DISOclust server was used for prediction of VIP1 disordered regions (S1 Fig) [25,32]. In the future, it would be interesting to test the structural similarities of protein orthologues in an attempt to reflect on their functionality within different phytosystems.

### Expression Profile of *HvVIP1* in barley infected by pathogens

**Fig 3:**
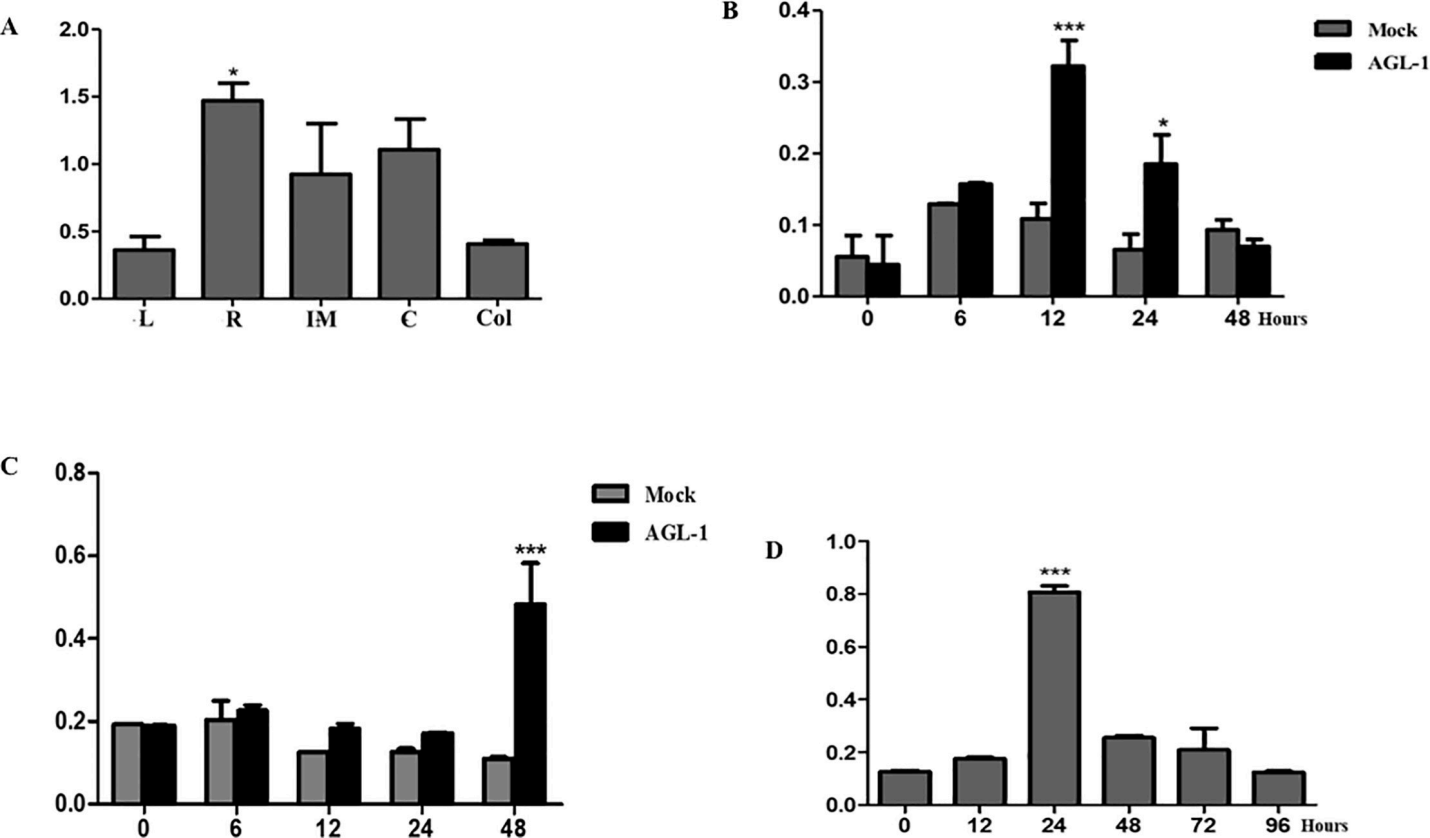
Time-dependent expression of VIP1 in barley cells. **(A)** *HvVIP1* expression profile in stress-free barley leaves (L), roots (R), immature embryos (IM), calli (C), and coleoptiles (Col). **(B)** *HvVIP1* significantly overexpressed (12^th^ hours) in cv. Golden promise calli after *Agrobacterium* (AGL-1 strain) infection. **(C)** *HvVIP1* induced significantly at the 48^th^ hour in cv. Martı calli treated with AGL-1 strain. **(D)** *HvVIP1* significantly overexpressed in *Fusarium*-inoculated barley roots 24 hours after treatment. (*) and (***) designate statistical significance. (*) for P < 0,05; and (***) for P < 0,001.

Gene expression examined in different barley tissues showed that *HvVIP1* is expressed in vascular tissues of plants like its counterpart members in the Group I bZIP proteins. Its expression profile in stress-free barley leaves, roots, immature embryos, coleoptiles and calli (Fig 3A) confirmed earlier findings regarding bZIP transcription factors’ role in vascular developmental and plant growth [16,28].

Infecting barley calli with the *Agrobacterium* strain AGL-1 led to significant overexpression of *HvVIP1* (a 3 fold increase in expression when compared to control/non-inoculated samples) just 12 hours after infection in cv. Golden Promise (susceptible to pathogen infection) (Fig 3B); while *HvVIP1* was induced significantly (a 5 fold increase) 48 hours after infection in calli of cv. Martı, a variety known for its pathogen-resistance [21] and tolerance to certain abiotic stresses [33] (Fig 3C). Furthermore, *HvVIP1* was induced in barley roots with a significant overexpression (8 fold) observed 24 hours after infection with the *Fusarium culmorum* F16 strain (Fig 3D).

Based on above-mentioned results, we concluded that VIP1 transcription factor seems to be a stress-induced agent in barley, not only reacting against *Agrobacterium*, but also responsive to a fungal pathogen.

Previously, VIP1 was described as a transcriptional regulator of stress-responsive genes in wounded and oxidative stress-treated plant cells, as well as a regulator of osmosensory signalling and mechanical stimuli in *Arabidopsis thaliana* [10,11, 34–36]. In 2008, a study [37] showed that some bZIP family members were upregulated in *Arabidopsis* by general elicitors of harpin and lipopolysaccharides (LPS), PAMPs that are unique to bacteria; whereas, later on, Lapham and colleagues [38] suggested that AtVIP1 may play a role in fungal (*Botrytis cinerea*) but not bacterial defense responses in *Arabidopsis* as it causes the upregulation of two defense related genes (MES1, LYK3) upon fungal attack. Interestingly enough, the recent findings [38] intersect with our results which indicate significant VIP1 induction in barley roots infected (after 24 hours) by another necrotrophic fungus, *Fusarium culmorum*. Taken together, those results strengthen the role of VIP1 in plant defence response but does not completely clarify it. The contrasting results regarding VIP1 role in defense against bacteria [38] remain to be elucidated by further comparative analyses in dicot and monocot plants.

### Expression analysis of *HvMPK1* and *HvPRs*

*HvMPK1* is significantly overexpressed in both barley cultivars after bacterial inoculation, particularly 6 hrs after infection (S2A and S2B Fig). Golden Promise calli are more susceptible to infection, with a higher *MPK1* induction of up to 500 folds, at the 12th hour. *HvMPK1* and *HvVIP1* showed a strong correlation, with a correlation coefficient of r = 0.90 (p ≤ 0.05) (Table 1) during *Agrobacterium* infection. *HvMPK1* was also significantly induced under fungal stress even relatively late after root infection (S2C Fig). The induction reaches its peak at 48 hours before it is subsided at 72 hours. No significant correlation was showed between *HvMPK1* and *HvVIP1* at any point during fungal infection (Table 1).

**Table 1:** Pearson correlation coefficients between *HvVIP1* and other stress responsive genes in barley calli.

Genes	*Agrobacterium* infection	*Fusarium* infection
***HvMPK3***	0.90*	NS
***HvPR1***	0.96**	0.77*
***HvPR3***	N/A	N/A
***HvPR4***	0.96**	Neg
***HvPR5***	N/A	N/A
***HvPR10***	0.95**	Neg

(NS) Not significant correlation; (Neg) Negative or downhill correlation; (N/A) Not available data. Significance coefficients

(*) P≤0.05, and

(**) P≤ 0.01

The strong correlation between *HvVIP1* and a barley MAPK family member protein, *HvMPK1* [22], under bacterial infection in barley cells simulates with earlier findings [11] stating that VIP1 is a component of the MAPK signal transduction pathway, regulating stress-dependent genes in a VRE-dependent manner in *Arabidopsis*. Indeed, the phosphorylation of VIP1 at Serine 79 residue leads to its nuclear transport in plant cells [10]. Thus, our results reflect on the validity of the proposed “MAPK->VIP1->VRE->gene activation” hypothesis in phytosystems other than the model organism *A*. *thaliana*.

On the other side, strongly induced *HvMPK1* in *Fusarium*-treated barley cells was no surprise at all as it reflects on its probable role as a member of stress-responsive MAPKs family member perceiving pathogen elicitors [39]. Even though the MAPK family has not been systematically studied in barley, some of its members are found involved in regulating stress responses citing among others but not limited to barley leaf rust caused by *Puccinia hordei* infection [22].

Gene expression analysis of *HvPR1*, *HvPR4* and *HvPR10* showed that these genes are induced by *Agrobacterium* infection in a time-dependent manner. Transcript accumulation was higher in *Agrobacterium*-infected calli than in *Fusarium*-infected roots (e.g. the induction of *HvPR4* in *Agrobacterium*-treated calli was up to 60 folds increased versus 20 folds only in *Fusarium*-inoculated roots). In addition, the number of transcripts was significantly higher in tolerant barley (Martı) compared to the sensitive genotype (cv. Golden Promise). The 24th and 48th hours after infection with *Agrobacterium* were critical for the timing of PR induction. In Martı calli, a strong significant correlation (r = 0.96) was detected between *HvVIP1* and *HvPR1* (Table 1), while no correlation was found for Golden Promise. We did not observe any detectable expression of *HvPR3* and *HvPR5* in *Agrobacterium*-infected barley callus. These genes were also slightly induced in *Fusarium*-infected roots.

Moreover, *HvVIP1* and *HvPR1* expression appeared to be harmonized in the Martı root infected with *F*. *culmorum*. Both genes are induced 24 hours after infection, as shown in S2D Fig. Indeed, a significant correlation between *HvVIP1* and *HvPR1* was detected with the Pearson test, revealing a relatively strong positive linear correlation between the two genes tested (r = 0.77).

In a recent study conducted upon VIP2 protein (another VIP protein), authors showed its regulatory role upon *NtPR1*, a stress-responsive gene, after fungal attack, in tobacco crops [40]. Thus, concluding to the fact that this VIP2-*NtPR1* upregulation might be behind Tobacco’s resistance to powdery mildew disease. The TaVIP2-*NtPR1* reflect on the HvVIP1-*HvPR1* upregulation model but within different pathosystem. The interrelationship between VIP proteins and PR genes should be investigated and their role in conferring plant resistance to pathogen attack (caused by different species of fungi e.g. *B*. *cinerea* [41]) must be elucidated.

Testing the VIP1- PRs interrelationship in barley tissues is an original work that lead to the detection of a strong correlation between VIP1 and each of the PRs (PR1, PR4, and PR10) (Table 1). However, this outcome was not the same within barley cultivars (Martı vs. Golden Promise) undergoing the same bacterial infection process. The different expression patterns of plant-defense involved factors among cultivars might be due to genotype-related “Susceptibility” to *Agrobacterium* [42]. Those results should be re-investigated and supported by further analysis (e.g. search for VRE elements in PR gene promoters, in-vitro protein-DNA binding assays, etc.).

## Material and methods

### Plant materials and growth conditions

Seeds of barley genotypes cv. Martı and cv. Golden Promise were provided by the Trakya Agricultural Research Institute (Turkey) and USDA (USA) respectively. The Martı cultivar is relatively pathogen- resistant [21], while the Golden Promise cultivar is known for its characteristic susceptibility to pathogens. Three leaf stage plantlets (18 days old) were generated in a hypodroponic system, supplemented with 1X Hoagland solution [43], and grown under controlled conditions (25°C day/night, 40–50% relative humidity, 8h light/16h dark photoperiod with a light intensity of 600 μMol m^-2^ s^-1^). Calli were generated from immature embryos sections, grown in the dark under controlled conditions, on MS medium supplemented with 2.5 mg/L Dicamba. Six to eight weeks old calli were selected carefully to proceed with.

### Isolation of total RNA and sequencing of HvVIP1 cDNA from barley

Total RNA isolation was performed according to a Trizol-based extraction from barley tissues. Full-length cDNAs of *H*. *vulgare* L. were obtained using the “Superscript First-Strand Synthesis System for RT-PCR” commercial kit (Invitrogen, #11904–018). VIP1 gene sequence was mapped through the NCBI Genbank tools, VIP1 transcripts were screened and amplified by PCR analysis using two sets of primers: [(i): F/ 5’ ACAGCGACTTCTCCTTCT 3’; R/ 5’ CCGAACTGTGATCGGTATG 3’; (ii): F/ 5’ TACCGGACTACGCCAAGA 3’; R/ 5’ ACCGAACTGTGATCGGTATG 3’]. Primers were designed in such a way to cover the whole gene coding region and were obtained according to the “Primer quest tools- IDT technologies” software. PCR conditions were 94°C for 3mins; 94°C for 30s; 58°C for 30s; and 72°C for 1min- 34 cycles in total with a 5 mins final elongation step (T100 Thermal Cycle, Bio-Rad, #1861096). Aliquots of individual PCR products (5 μL) were loaded on 1% (w/v) agarose gel and visualized with ethidium bromide staining under UV light. VIP1 cDNA fragments were then cloned following the manufacturer’s instructions (Thermo Scientific InsTAclone PCR Cloning Kit, #K1213). For sequencing, BigDye Terminator v3.1 Cycle Sequencing Kits (Applied Biosystems (QVs>20) were used, and GeneMapper IDx v4 software was employed for chromatogram analysis. ORFs and prediction transcription start sites (TSS) were identified using the ORF Finder tool (http://www.ncbi.nlm.nih.gov/gorf.html).

### Nucleotide sequence and bioinformatics analysis

Structural similarities between VIP1 orthologues were identified through comparative analyses performed using bioinformatics tools. Full-length sequences of the VIP1-like Proteins from Arabidopsis (NP_564486.1), Barley (BAJ96285.1) and Rice (XP_015618202.1) were initially submitted to the IntFOLD server [29] to obtain 3D models with quality estimates [30], template structures, functional information, structural domain predictions and disorder prediction profiles. Sequences were searched against the Pfam database [44] to obtain sequence domain information. The sub-sequences of the ordered regions, predicted using DISOclust [25], were then taken and submitted to the IntFOLD server to obtain higher quality 3D models [30].

For *HvVIP1* gene prediction, the full-length cloned VIP1 nucleotide sequence was searched against the barley genome database using BLAST (https://blast.ncbi.nlm.nih.gov/Blast.cgi) (morexGenes, Accession No: MLOC_71198). Details of the exon and intron gene structure were deduced via sequence alignment assay using the ClustalW2 program (EMBL-EBI/MUSCLE). Phylogenetic analyses were conducted based on the JTT matrix-based model [45] of the Molecular Evolutionary Genetics Analysis software (MEGA7) (http://www.megasoftware.net/mega4/mega.html). Ten amino acid sequences (full-length VIP1 protein sequences) derived from complete coding sequences identified in the genomes of various monocotyledonous (i.e. wheat, maize, rice, etc.) and dicotyledonous species (i.e. *Arabidopsis*) were compared for their evolutionary relationships and trees were drawn to scale, with branch lengths measured as the number of substitutions per site [46]. MEGA 7 tool using Maximum Likelihood statistical methods were used for this matter.

### Plant stress applications

Colonies of *A*. *tumefaciens* strain AGL-1 were grown on LB medium (10 g/L tryptone, 10 g/L yeast extract, 5 g/L NaCl, pH 7.0), supplemented with 20 mg/L rifampicin and 200 mg/L carbenicillin. Colonies reaching a certain log phase (0.8 <OD_600_ <1) were harvested and the pelleted cells were re-suspended in liquid half-strength MS (Murashige and Skoog) inoculation medium (IM) containing 10 mM MES [2-(N-morpholine)-ethane sulphonic acid], 200 μM acetosyringone, 10g/ L glucose, 1g/L pluronic acid, and 2mM CaCl_2_, where they grew to a final optical density of OD_600_ = 1 [47]. Prior to infection, AGL-1 colonies were left to rest in the IM, for 1 hour, in the dark. Eight-week-old fresh embryogenic calli from both cv. Martı (pathogen-resistant) and cv. Golden Promise (pathogen-sensitive) were soaked in the inoculation medium for 30 mins, in the dark, at room temperature [48,49], then left to dry on sterile papers before being transferred into a co-cultivation medium (MS medium supplemented with Dicamba 2.5mg/L, 500μM Acetosyringone, and 800 mg/L freshly prepared L-cystein). Infected calli were laid on co-cultivation medium for 6, 12, 24, and 48 hours, in the dark. Mock-inoculated calli were submerged in an *Agrobacterium*-free Inoculation Medium (IM Δ AGL-1). They went through the same procedure as the bacterium-inoculated calli. 0 hours represents the point in time before any treatment is performed. Harvested calli from different time points were ground up and preserved at -80°C, prior to any further molecular examination. For the model of *Fusarium culmorum* infection, the highly pathogenic isolate *F*. *culmorum* (F16, also known as F16-5) [50] was used to infect barley roots. The fungal growth and development were performed according to the procedure outlined in [51]. Basically, the strain F16 were cultured on Petri dishes containing Potato Dextrose Agar medium (PDA) and incubated for 7 days under controlled conditions of 25°C for 12/12 h of a day/night cycle and 50% relative humidity, prior to barley root infection. For infection, PDA medium discs (5–6 mm diameter) of the strain F16 were collected and then fixed upon the plantlet’s main root in such a way that the mycelium will be in direct contact with it, at a depth of approximately 1 cm underneath the seed (3-day old plant seedlings). In the control treatments, sterile PDA medium discs were placed on the roots. Samples were collected from both groups (control and infected) at several time points (i.e. 12, 24, 48, 72, and 96 hours). The assay was carried out in a controlled growth chamber (Angelantoni EHC, Massa Martana, PG, Italy) with a light intensity of 600 μmol m^-2^ s^−1^ at 25°C, 40% relative humidity and 16/8 h of day/night cycle. The pathogenic inoculation with F16-PDA medium disks of barley roots was already tested and its efficiency was proved according to [21].

### Quantitative real time PCR

The total RNA, extracted from barley tissues, was purified and first-strand cDNA fragments of *HvVIP1*, *HvMPK1*, and *HvPR* genes from *Hordeum vulgare* L. were synthesized, as described earlier. The forward and reverse primers of the genes tested are given in Table 2. Primers were designed through IDT tools, and real-time PCR was performed on a Roche Light Cycler 480. The efficiency of these primers was investigated by applying primer melting curve analysis and gel electrophoresis; both results indicated that each primer pair gave a specific and unique PCR product. Reactions and data analysis were performed as described in [21]. Each reaction consisted of a total volume of 10 μL containing 100 ng of cDNA, 1 U of GoTaq DNA polymerase (Promega, M830), 0.2 mM dNTP, 2.5 mM MgCl_2_, 0.2 μM of each Forward and Reverse primers specific for each gene (Table 2) and 0.5 μL of 20× Eva Green dye (Biotium, 31000). Each experiment was performed in duplicate and with three biological replicates.

**Table 2:** Primer sequences for gene expression analysis by qPCR.

Gene	Accession No	Primers	Annealing Temperature (°C)	Amplicon (bp)
*HvVIP1*	AK365082	5’-GAGAGCTCAAACTCCGGTTG -3’ 5’-GAACTTGACCTGCCGCTATC -3’	60	114
*HvPR1*	X74939	5’-CGCCGGGAATGTTGTTGGAC-3’ 5’-ACGAGCGTGCATGAATCCCA-3’	60	126
*HvPR3*	X78672	5’-AAGGCCACGTCTCCACCCTA-3’ 5’CAGGTCCGGGTTGCTCACAA-3’	58	114
*HvPR4*	Y10814	5’-GCCAAGATCGACACCAACGG-3’ 5’-CAGTCCGAGCTGCTCACTCA-3’	60	150
*HvPR5*	AJ276225	5’-ACGACATCTCGGTTATCGACG-3’ 5’-TTATTGCCACTGCAGGCGT-3’	60	151
*HvPR10*	AY220734	5’-GCGGCTAGGGTGTTCAAGAC-3’ 5’-CCTGAGCTTCGCCACACAAG-3’	60	123
*HvMPK1*	AK376245	5’ -GGTCACCGCCAAGTACAA -3’ 5’ -AAGGCGTTGGCGATCTT -3’	59	123
*HvACT*	AK362208	5’-CGTGTTGGATTCTGGTGATG -3’ 5’-AGCCAC ATATGCGAGCTTCT -3’	60	208

## Conclusion

Our work provides insight into some of the structural and functional features of VIP1 and its orthologues in cereal species that are known to be recalcitrant to *Agrobacterium*-mediated transformation when compared to the host crop *Arabidopsis*, however it is clear that further studies are required. We have discovered that *HvVIP1* is expressed in barley cells under normal conditions while pathogenic infections induce its transcription temporarily. In addition, there is a transcriptional link between *HvVIP1* expression and the induction of *HvPR* genes. In *Arabidopsis*, a rapid activation of MPK3 upon stress is followed by VIP1 phosphorylation and nuclear translocation within minutes, as has been shown in earlier studies. Our study confirmed the substantial expression of a MAPK protein, HvMPK1, for the first time in relation to both *Agrobacterium* and *Fusarium*-infections. This finding clearly puts MAPK signal transduction as a key factor in the response to stress and subsequent tolerance in barley cells. In the future work, it would be interesting to study the expression patterns of other MPK genes in barley as well as investigate in-depth the regulatory role of *HvVIP1* upon stress-responsive genes, such as PRs.

## Supporting information

S1 Fig Structural comparison of VIP1-like proteins from *Arabidopsis* and barley. (**A)** 3D model using the full-length *Arabidopsis* sequence obtained from the IntFOLD server (McGuffin *et al*., 2015). The model is coloured according to the ModFOLD6 (Maghrabi *et al*., 2017) predicted per-residue accuracy using a temperature spectrum from blue-red (blue, high confidence and ordered; red, low confidence & disordered). (**B)** 3D model using the full-length *H*. *vulgare* sequence obtained from the IntFOLD server. (**C)** Intrinsic disorder prediction profile of the full-length barley sequence, from DISOclust (McGuffin, 2008).
(TIFF)

S2 Fig ***HvMPK1* expression profile in (A)** *Agrobacterium-infected* cv. Golden promise; **(B)** in *Agrobacterium*-infected cv. Martı calli; **(C)** in *Fusarium*-inoculated cv. Martı roots. **(D)** Schematic representation of the correlation pattern (r = 0.77) between *HvVIP1* and *HvPR1* in *Fusarium*-inoculated barley roots. (*) and (***) designate statistical significance. (*) for P < 0,05; and (***) for P < 0,001.
(TIFF)
