# Supplementary figures and images for: Characterisation of HvVIP1 and expression profile analysis of stress response regulators in barley under Agrobacterium and Fusarium infections
Source: PLoS One. 2019 Jun 14;14(6):e0218120. doi: 10.1371/journal.pone.0218120 (PMC6570034; doi:10.1371/journal.pone.0218120)

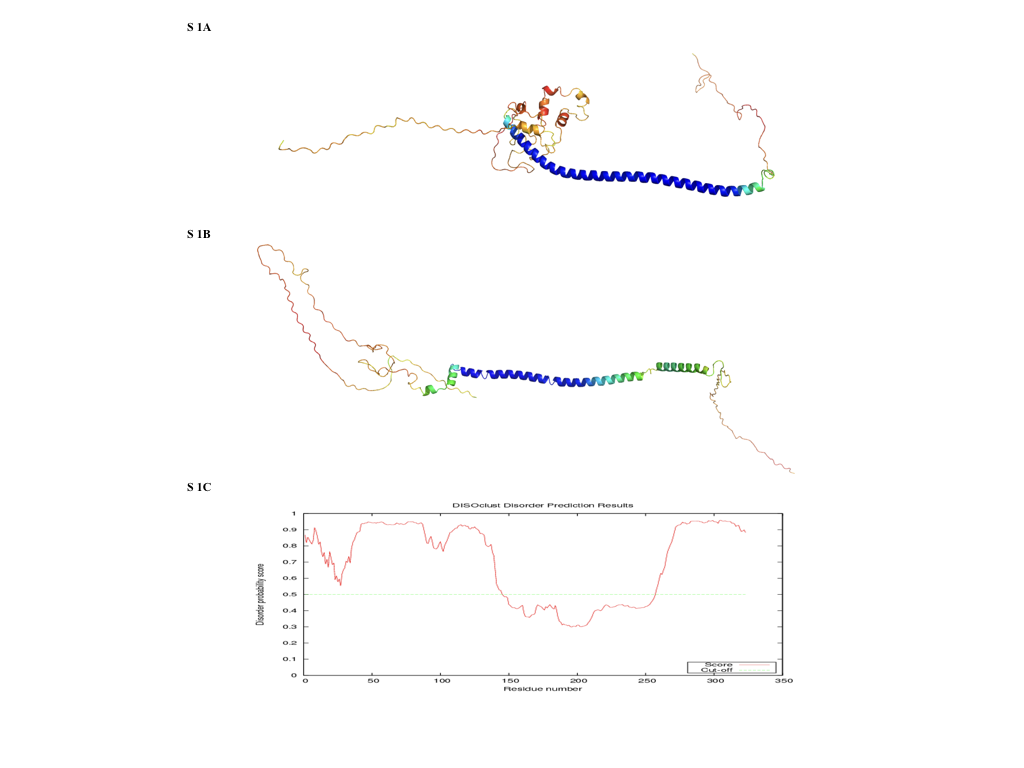

Supplement: S1 Fig — (A) 3D model using the full-length Arabidopsis sequence obtained from the IntFOLD server (McGuffin et al., 2015). The model is coloured according to the ModFOLD6 (Maghrabi et al., 2017) predicted per-residue accuracy using a temperature spectrum from blue-red (blue, high confidence and ordered; red, low confidence & disordered). (B) 3D model using the full-length H. vulgare sequence obtained from the IntFOLD server. (C) Intrinsic disorder prediction profile of the full-length barley sequence, from DISOclust (McGuffin, 2008). (TIFF) [file pone.0218120.s001.tiff]

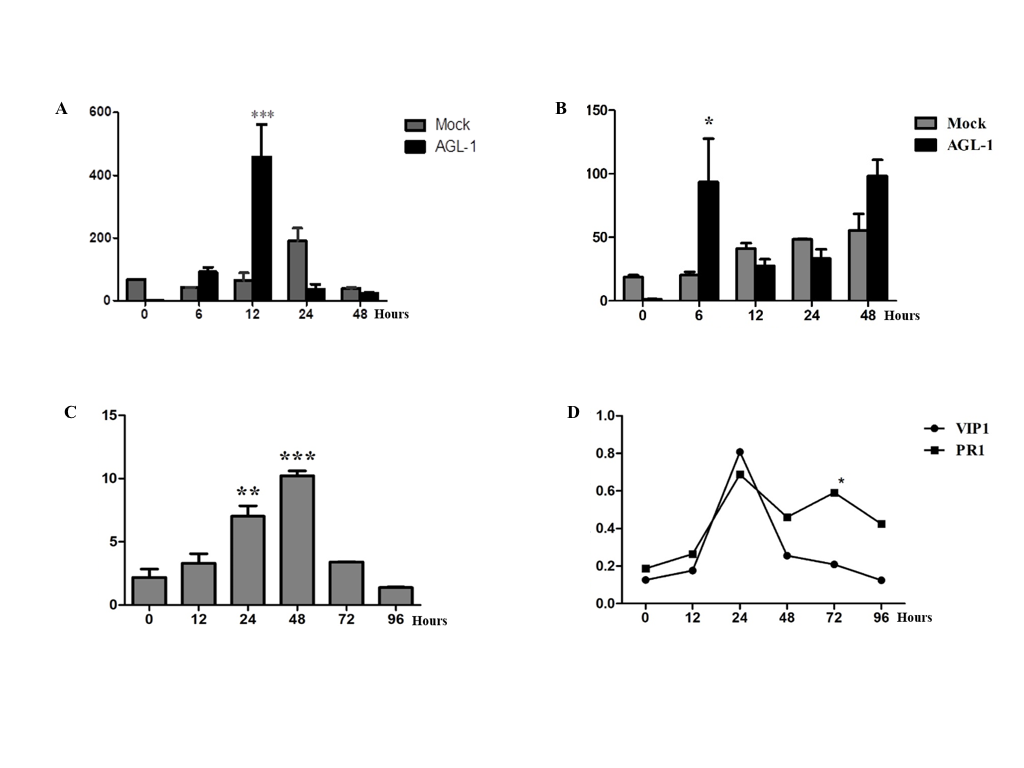

Supplement: S2 Fig — HvMPK1 expression profile in (A) Agrobacterium-infected cv. Golden promise; (B) in Agrobacterium-infected cv. Martı calli; (C) in Fusarium-inoculated cv. Martı roots. (D) Schematic representation of the correlation pattern (r = 0.77) between HvVIP1 and HvPR1 in Fusarium-inoculated barley roots. (*) and (***) designate statistical significance. (*) for P < 0,05; and (***) for P < 0,001. (TIFF) [file pone.0218120.s002.tiff]
